# Outcomes and Mortality Among Adults Hospitalized With COVID-19 at US Medical Centers

**DOI:** 10.1001/jamanetworkopen.2021.0417

**Published:** 2021-03-05

**Authors:** Ninh T. Nguyen, Justine Chinn, Jeffry Nahmias, Sarah Yuen, Katharine A. Kirby, Sam Hohmann, Alpesh Amin

**Affiliations:** 1 Department of Surgery, University of California, Irvine Medical Center, Orange; 2 Department of Statistics, University of California, Irvine, Orange; 3 Vizient, Centers for Advanced Analytics, Chicago, Illinois; 4 Department of Medicine, University of California, Irvine Medical Center, Orange

## Abstract

This cohort study assesses characteristics and outcomes among adults hospitalized with coronavirus disease 2019 (COVID-19) at US medical centers as well as COVID-19–related mortality over the initial 6 months of the pandemic.

## Introduction

Coronavirus disease 2019 (COVID-19) originally emerged from China and has since spread globally, with almost 14 million confirmed cases and more than 260 000 deaths in the US as of December 1, 2020.^1^ To date, there have been regional reports on outcomes among patients who developed serious symptoms requiring hospitalization.^2,3,4,5^ The objectives of our study were to examine the characteristics and outcomes among adults hospitalized with COVID-19 at US medical centers and analyze changes in mortality over the initial 6-month period of the pandemic.

## Methods

The data for this cohort study were obtained from the Vizient clinical database (Clinical Data Base/Resource Manager), which is an administrative, clinical, and financial database of more than 650 academic centers and their affiliates from 47 US states. Approval for the use of the data was obtained from Vizient and from the institutional review board of the University of California, Irvine, as exempted status because patient data are deidentified. This study followed the Strengthening the Reporting of Observational Studies in Epidemiology (STROBE) reporting guideline.

Discharge records of adults 18 years or older who had received a diagnosis of COVID-19 and were admitted to the hospital between March 1 and August 31, 2020, were reviewed. Patients with COVID-19 were identified using *International Statistical Classification of Diseases and Related Health Problems, Tenth Revision* code U07.1. The primary outcome was in-hospital mortality, which was analyzed according to the month of admission and age group and in a subgroup of patients requiring intensive care unit (ICU) admission. Secondary outcomes included length of hospital stay, length of ICU stay, and median cost of ICU stay vs non-ICU stay. Survival probabilities by length of stay were plotted according to month of admission and patient age group. The Cochran-Armitage test was used to assess the linear trend in mortality proportions over time. Statistical significance was set at α = .05 for 2-sided *P* values. Analyses were performed using Stata, version 16 (StataCorp LLC).

## Results

Among 192 550 adults hospitalized with COVID-19 who were discharged from 555 US medical centers, 101 089 (52.5%) were men, 83 567 (43.3%) were White, and 125 543 (65.2%) had Medicare or Medicaid insurance. The most common comorbidities included hypertension (118 418 [61.5%]), diabetes (73 939 [38.4%]), and obesity (52 759 [27.4%]).

Of patients in this cohort, 55 593 (28.9%) were admitted to the ICU, 26 221 (13.6%) died during the index hospitalization, and 5839 (3.0%) were transferred to hospice care (Table). In-hospital mortality increased in association with increasing age; 179 of 12 644 patients (1.4%) aged 18 to 29 years died, and 8277 of 31 135 patients (26.6%) 80 years or older died. Of the patients admitted to the ICU, 15 431 of 55 593 (27.8%) died (Figure, A). The median hospital length of stay among patients who were not admitted to the ICU was 6 days (interquartile range [IQR], 3-8 days), with a median cost per admission of $10 520 (IQR, $8031-$14 550). The median hospital length of stay for those admitted to the ICU was 15 days (IQR, 6-20 days), with a median cost per admission of $39 825 (IQR, $25 763-$56 804). There was a significant reduction in mortality over the course of the 6-month period, with the highest mortality in March (3657 of 16 517 patients died [22.1%]); mortality decreased each month until the end of the study period in August (1154 of 17 776 patients died [6.5%]) (χ^2^ for trend, 3592.3; *P* < .001) (Figure, B).

**Table.** Clinical Outcomes Among Adults Hospitalized With Coronavirus Disease 2019 at 555 US Medical Centers

Outcome	Patients, No./total No. (%)
In-hospital	
Mortality or discharged to hospice	32 060/192 550 (16.6)
Mortality	26 221/192 550 (13.6)
In-hospital mortality by mo of admission	
March	3657/16 517 (22.1)
April	11 880/65 475 (18.1)
May	4101/34 071 (12.0)
June	2204/24 088 (9.1)
July	3192/34 482 (9.2)
August	1154/17 776 (6.5)
In-hospital mortality by age, y	
18-29	179/12 644 (1.4)
30-39	471/17 172 (2.7)
40-49	1185/22 888 (5.2)
50-59	3047/34 532 (8.8)
60-69	5921/40 344 (14.7)
70-79	7141/33 835 (21.1)
≥80	8277/31 135 (26.6)
Length of stay, median (IQR), d	
Without ICU stay	6 (3-8)
With ICU stay	15 (6-20)
ICU admission	55 593/192 550 (28.9)
Median cost of stay, median (IQR), $	
Without ICU stay	10 520 (8031-14 550)
With ICU stay	39 825 (25 763-56 804)

Abbreviations: ICU, intensive care unit; IQR, interquartile range.

**Figure.**
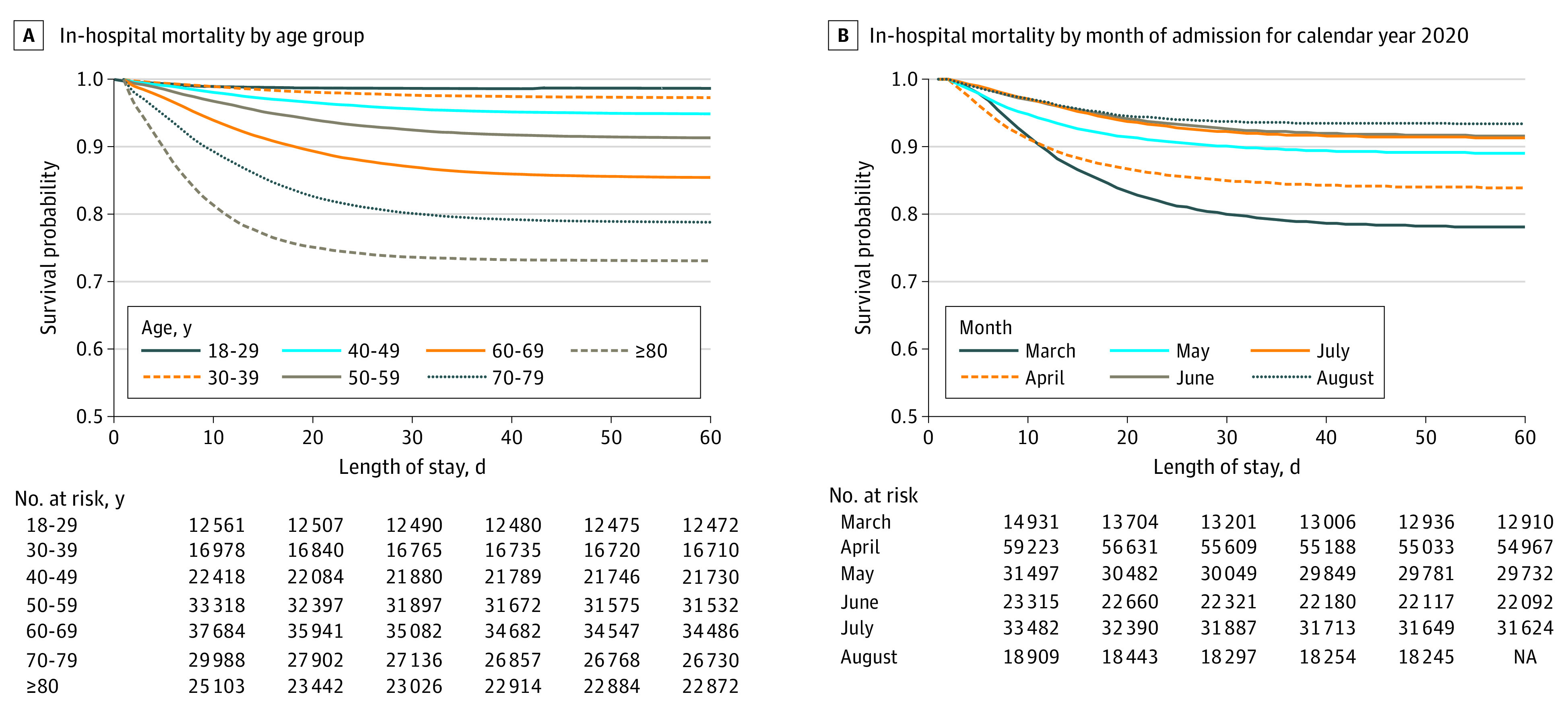
In-Hospital Mortality Among Adults With Coronavirus Disease 2019 (COVID-19) Who Were Discharged From 555 US Medical Centers by Age Group and Month of Admission

## Discussion

This cohort study of patients with COVID-19 who were admitted to US medical centers revealed high in-hospital mortality of 13.6%. However, over the course of the pandemic, there was a reduction in mortality of more than 15 percentage points between March (22.1%) and August (6.5%). The in-hospital mortality in the current study was similar to that reported in other published US studies (15.3%-24.5%).^2,3,4,5^ Mortality increased in association with increasing age. Patients 80 years or older represented the age group with the highest mortality. There are several limitations to this retrospective study, including misclassification and accuracy of coding and missing data. Management of COVID-19 is rapidly changing, and this study did not compare treatment modalities; radiologic and laboratory clinical findings were not available. It is also possible that at the beginning of the pandemic, COVID-19 was underdiagnosed owing to the lack of widespread testing availability in the US; however, we believe that for most of the study period, COVID-19 diagnoses were accurately reflected with increased availability of testing. Despite these limitations, this study provides data on characteristics and outcomes in, to our knowledge, the largest US cohort of hospitalized COVID-19 adults to date; identified subgroups of patients with higher mortality; and determined mortality over time (from March 1 to August 31, 2020) at 555 US medical centers.
